# (2*E*)-1-(3-Bromo­phen­yl)-3-(4,5-dimeth­oxy-2-nitro­phen­yl)prop-2-en-1-one

**DOI:** 10.1107/S1600536810041292

**Published:** 2010-10-23

**Authors:** Jerry P. Jasinski, Ray J. Butcher, C. S. Chidan Kumar, H. S. Yathirajan, A. N. Mayekar

**Affiliations:** a Department of Chemistry, Keene State College, 229 Main Street, Keene, NH 03435-2001, USA; b Department of Chemistry, Howard University, 525 College Street NW, Washington DC 20059, USA; c Department of Studies in Chemistry, University of Mysore, Manasagangotri, Mysore 570 006, India; d Sequent Scientif Limited, New Mangalore 57 011, India

## Abstract

In the title compound, C_17_H_14_BrNO_5_, the dihedral angle between the 3-bromo-substituted benzene ring and the 4,5-dimeth­oxy-2-nitro-phenyl ring is 15.2 (1)°. The dihedral angles between the mean plane of the propenone group and the mean planes of the 3-bromo-substituted benzene and 4,5-dimeth­oxy-2-nitro­phenyl rings are 6.9 (6) and 20.5 (5)°, respectively. Weak inter­molecular C—H⋯O inter­actions contribute to crystal stability and π–π inter­actions [centroid–centroid distances = 3.7072 (18) and 3.6326 (18) Å] are also observed.

## Related literature

For the biological activity of chalcones, see: Liu *et al.* (2003 ▶); Nielson *et al.* (1998 ▶); Rajas *et al.* (2002 ▶); Dinkova-Kostova *et al.* (1998 ▶). For their non-linear optical properties, see: Goto *et al.* (1991 ▶); Uchida *et al.* (1998 ▶);Tam *et al.* (1989 ▶); Indira *et al.* (2002 ▶); Sarojini *et al.* (2006 ▶). For the effect of bulky substit­uents on the spontaneous polarization of non-centrosymmetric crystals, see: Fichou *et al.* (1988 ▶). For the influence of the steric effect of the substituent on the mol­ecular hyperpolarizability, see: Cho *et al.* (1996 ▶). For related structures, see: Butcher *et al.* (2007*a* ▶,*b* ▶,*c* ▶); Jasinski *et al.* (2010*a* ▶,*b* ▶,*c* ▶,*d* ▶,*e* ▶); Dutkiewicz *et al.* (2010 ▶); Kant *et al.* (2009 ▶); Yathirajan *et al.* (2007 ▶).
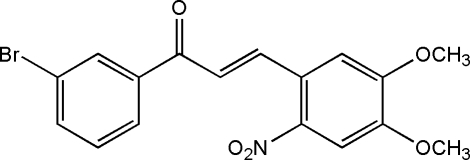


## Experimental

### 

#### Crystal data

C_17_H_14_BrNO_5_
*M* *_r_* = 392.20
Orthorhombic, 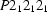

*a* = 6.8547 (2) Å
*b* = 8.3205 (2) Å
*c* = 27.1509 (6) Å
*V* = 1548.54 (7) Å^3^
*Z* = 4
Cu *K*α radiation
μ = 3.88 mm^−1^
*T* = 123 K
0.55 × 0.12 × 0.06 mm

#### Data collection

Oxford Diffraction Xcalibur Diffractometer with Ruby Gemini detector
Absorption correction: multi-scan (*CrysAlis RED*; Oxford Diffraction, 2007 ▶) *T*_min_ = 0.490, *T*_max_ = 1.000
9914 measured reflections
3069 independent reflections
3011 reflections with *I* > 2σ(*I*)
*R*_int_ = 0.040

#### Refinement

*R*[*F* ^2^ > 2σ(*F* ^2^)] = 0.032
*wR*(*F* ^2^) = 0.086
*S* = 1.07
3069 reflections
219 parameters
H-atom parameters constrained
Δρ_max_ = 0.74 e Å^−3^
Δρ_min_ = −0.42 e Å^−3^
Absolute structure: Flack (1983 ▶), 1228 Friedel pairs
Flack parameter: 0.08 (2)

### 

Data collection: *CrysAlis PRO* (Oxford Diffraction, 2007 ▶); cell refinement: *CrysAlis PRO*; data reduction: *CrysAlis RED*; program(s) used to solve structure: *SHELXS97* (Sheldrick, 2008 ▶); program(s) used to refine structure: *SHELXL97* (Sheldrick, 2008 ▶); molecular graphics: *SHELXTL* (Sheldrick, 2008 ▶); software used to prepare material for publication: *SHELXTL*.

## Supplementary Material

Crystal structure: contains datablocks I. DOI: 10.1107/S1600536810041292/lx2178sup1.cif

Structure factors: contains datablocks I. DOI: 10.1107/S1600536810041292/lx2178Isup2.hkl

Additional supplementary materials:  crystallographic information; 3D view; checkCIF report

## Figures and Tables

**Table 1:** Hydrogen-bond geometry (Å, °)

*D*—H⋯*A*	*D*—H	H⋯*A*	*D*⋯*A*	*D*—H⋯*A*
C16—H16*A*⋯O5^i^	0.98	2.46	3.383 (3)	157
C17—H17*B*⋯O3^ii^	0.98	2.48	3.116 (4)	123

Symmetry codes: (i) 
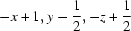
; (ii) 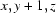.

**Fig. 1.**
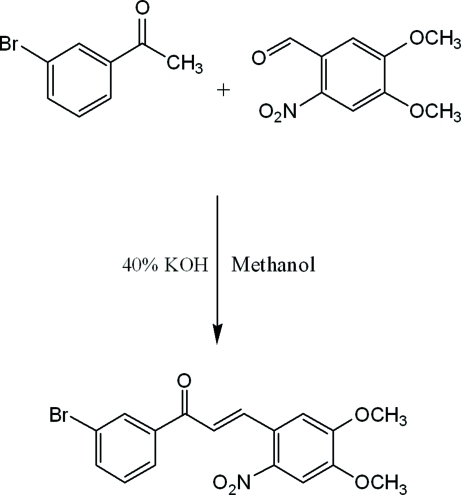
Reaction scheme for the title compound.

**Fig. 2.**
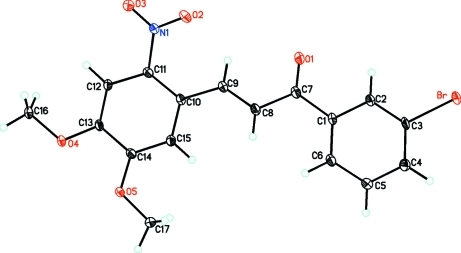
Molecular structure of the title compound showing the atom labeling scheme and 50% probability displacement ellipsoids.

**Fig. 3.**
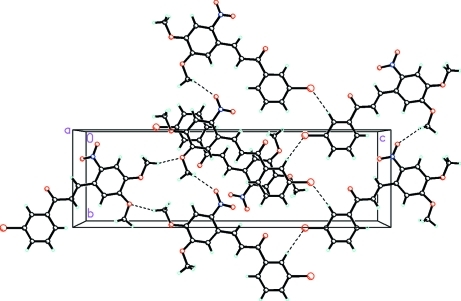
Packing diagram of the title compound viewed down the a axis. Dashed lines indicate weak intermolecular C—H···O hydrogen bond interactions.

## supplementary crystallographic information

### Comment

Chalcones have displayed an impressive array of biological activities, among which antimalarial (Liu *et al.*, 2003), antiprotozoal (Nielson *et al.*, 1998), nitric oxide inhibition (Rajas *et al.*, 2002) and anticancer activities (Dinkova-Kostova *et al.*, 1998) have been cited in the literature. Among several organic compounds reported for non-linear optical (NLO) properties, chalcone derivatives are notable materials for their excellent blue-light transmittance and good crystallizability. They provide the necessary configuration to show NLO properties, with two planar rings connected through a conjugated double bond (Goto *et al.*, 1991; Uchida *et al.*, 1998; Tam *et al.*, 1989; Indira *et al.*, 2002, Sarojini *et al.*, 2006). Substitution on either of the benzene rings greatly influences the non-centrosymmetric crystal packing. It is speculated that, in order to improve the activity, more bulky substituents should be introduced to increase the spontaneous polarization of non-centrosymmetric crystals (Fichou *et al.*, 1988). The molecular hyperpolarizability is strongly influenced, not only by the electronic effect, but also by the steric effect of the substituent (Cho *et al.*, 1996). The crystal structure studies of 2,3-dibromo-1-(2,4-dichlorophenyl)-3-(4,5-dimethoxy-2-nitrophenyl) propan-1-one (Yathirajan *et al.*, 2007); (2*E*)-1-(4-methylphenyl)-3-(4-nitrophenyl)prop-2-en-1-one (Butcher *et al.*, 2007*a*); (*E*)-3-(4-fluorophenyl)-1-(4-methylphenyl)prop-2-en-1-one (Butcher *et al.*, 2007*b*); (2*E*)-3-(2-bromo-5-methoxyphenyl)-1-(2,4-dichlorophenyl) prop-2-en-1-one (Butcher *et al.*, 2007*c*); (*E*)-3-(4-bromophenyl)-1-(3,4-dichlorophenyl)prop-2-en-1-one (Kant *et al.*, 2009); (2E)-3-(4-bromophenyl)-1-(3-chlorophenyl) prop-2-en-1-one (Jasinski *et al.*, 2010*a*); (2*E*)-1-(4-bromophenyl)-3-(4-fluorophenyl)prop-2-en-1-one (Dutkiewicz *et al.*, 2010); (2E)-1-(2-bromophenyl)-3-(4-chlorophenyl) prop-2-en-1-one (Jasinski *et al.*, 2010*b*); (2*E*)-1-(2-bromophenyl)-3-(4-methoxyphenyl)prop-2-en-1-one (Jasinski *et al.*, 2010*c*); (2*E*)-1-(2-bromophenyl)-3- (3,4,5-trimethoxyphenyl)prop-2-en-1-one (Jasinski *et al.*, 2010*d*) and (2*E*)-1-(2-bromophenyl)-3-(4-bromophenyl)prop-2-en-1-one (Jasinski *et al.*, 2010*e*) have been reported. In continuation of our work on chalcones, the present paper reports the synthesis and crystal structure of a new chalcone, C_17_H_14_BrNO_5_.

In the title compound the dihedral angle between the 3-bromo-substituted benzene ring and the 4,5-dimethoxy-2-nitro-phenyl ring is 15.2 (1)° (Fig. 2). The dihedral angles between the mean plane of the propenone group and the mean planes of the 3-bromo-substituted benzene and 4,5-dimethoxy-2-nitro-phenyl rings is 6.9 (6)° and 20.5 (5)°, respectively. While no classic hydrogen bonds are observed, weak intermolecular C—H···O (Table 1, Fig. 3) hydrogen bond interactions contribute to crystal stability.

### Experimental

1-(3-Bromophenyl)ethanone (1.99 g, 0.01 mol) was mixed with 4,5-dimethoxy-2-nitrobenzaldehyde (2.11 g, 0.01 mol) and dissolved in methanol (30 ml). To this, 3 ml of KOH (40%) was added and the reaction mixture was stirred for 6 h (Fig. 1). The resulting crude solid was filtered, washed successively with distilled water and finally recrystallized from ethanol (95%) to give the pure chalcone. Pale yellow, small needle shaped crystals suitable for X-ray diffraction studies were grown by the slow evaporation of the dimethylformamide solution at room temperature (m.p.: 409–411 K).

### Refinement

The parameters of all the H atoms have been constrained within the riding atom approximation. C—H bond lengths were constrained to 0.95 or 0.98 Å for aryl or methyl H atoms, *U*_iso_(H) = 1.18–1.22*U*_eq_(C_aryl_); *U*_iso_(H) = 1.59–1.51*U*_eq_(C_methyl_).

### Crystal data

**Table:**

C_17_H_14_BrNO_5_	*F*(000) = 792
*M**_r_* = 392.20	*D*_x_ = 1.682 Mg m^−^^3^
Orthorhombic, *P*2_1_2_1_2_1_	Cu *K*α radiation, λ = 1.54178 Å
Hall symbol: P 2ac 2ab	Cell parameters from 8339 reflections
*a* = 6.8547 (2) Å	θ = 4.9–74.0°
*b* = 8.3205 (2) Å	µ = 3.88 mm^−^^1^
*c* = 27.1509 (6) Å	*T* = 123 K
*V* = 1548.54 (7) Å^3^	Needle, colorless
*Z* = 4	0.55 × 0.12 × 0.06 mm

### Data collection

**Table:**

Oxford Diffraction Xcalibur Diffractometer with Ruby Gemini detector	3069 independent reflections
Radiation source: Enhance (Cu) X-ray Source	3011 reflections with *I* > 2σ(*I*)
graphite	*R*_int_ = 0.040
Detector resolution: 10.5081 pixels mm^-1^	θ_max_ = 74.1°, θ_min_ = 5.6°
ω scans	*h* = −8→5
Absorption correction: multi-scan (*CrysAlis RED*; Oxford Diffraction, 2007)	*k* = −9→10
*T*_min_ = 0.490, *T*_max_ = 1.000	*l* = −32→33
9914 measured reflections	

### Refinement

**Table:**

Refinement on *F*^2^	Secondary atom site location: difference Fourier map
Least-squares matrix: full	Hydrogen site location: inferred from neighbouring sites
*R*[*F*^2^ > 2σ(*F*^2^)] = 0.032	H-atom parameters constrained
*wR*(*F*^2^) = 0.086	*w* = 1/[σ^2^(*F*_o_^2^) + (0.0497*P*)^2^ + 1.5041*P*] where *P* = (*F*_o_^2^ + 2*F*_c_^2^)/3
*S* = 1.07	(Δ/σ)_max_ = 0.003
3069 reflections	Δρ_max_ = 0.74 e Å^−^^3^
219 parameters	Δρ_min_ = −0.42 e Å^−^^3^
0 restraints	Absolute structure: Flack (1983), 1228 Friedel pairs
Primary atom site location: structure-invariant direct methods	Flack parameter: 0.08 (2)

### Special details

**Table:**

Geometry. All e.s.d.'s (except the e.s.d. in the dihedral angle between two l.s. planes) are estimated using the full covariance matrix. The cell e.s.d.'s are taken into account individually in the estimation of e.s.d.'s in distances, angles and torsion angles; correlations between e.s.d.'s in cell parameters are only used when they are defined by crystal symmetry. An approximate (isotropic) treatment of cell e.s.d.'s is used for estimating e.s.d.'s involving l.s. planes.
Refinement. Refinement of *F*^2^ against ALL reflections. The weighted *R*-factor *wR* and goodness of fit *S* are based on *F*^2^, conventional *R*-factors *R* are based on *F*, with *F* set to zero for negative *F*^2^. The threshold expression of *F*^2^ > σ(*F*^2^) is used only for calculating *R*-factors(gt) *etc*. and is not relevant to the choice of reflections for refinement. *R*-factors based on *F*^2^ are statistically about twice as large as those based on *F*, and *R*- factors based on ALL data will be even larger.

### Fractional atomic coordinates and isotropic or equivalent isotropic displacement parameters (Å^2^)

**Table:**

	*x*	*y*	*z*	*U*_iso_*/*U*_eq_	
Br	0.59396 (5)	0.54840 (4)	0.757218 (10)	0.02935 (11)	
O1	0.5372 (4)	0.1224 (3)	0.60992 (8)	0.0329 (6)	
O2	0.7701 (4)	−0.1958 (3)	0.50347 (8)	0.0314 (5)	
O3	0.6537 (4)	−0.3581 (3)	0.44871 (9)	0.0332 (6)	
O4	0.5820 (4)	0.0031 (2)	0.30190 (7)	0.0240 (4)	
O5	0.5346 (4)	0.2869 (3)	0.33757 (7)	0.0258 (5)	
N1	0.6855 (4)	−0.2223 (3)	0.46423 (10)	0.0227 (5)	
C1	0.5862 (5)	0.4042 (3)	0.61065 (10)	0.0200 (5)	
C2	0.5815 (5)	0.4043 (3)	0.66260 (10)	0.0227 (6)	
H2A	0.5685	0.3064	0.6803	0.027*	
C3	0.5960 (4)	0.5491 (4)	0.68724 (9)	0.0229 (5)	
C4	0.6120 (5)	0.6943 (4)	0.66260 (11)	0.0247 (6)	
H4A	0.6189	0.7925	0.6804	0.030*	
C5	0.6179 (5)	0.6939 (4)	0.61128 (11)	0.0246 (6)	
H5A	0.6296	0.7925	0.5938	0.030*	
C6	0.6065 (4)	0.5491 (4)	0.58544 (10)	0.0230 (5)	
H6A	0.6126	0.5494	0.5505	0.028*	
C7	0.5701 (5)	0.2433 (4)	0.58575 (11)	0.0233 (6)	
C8	0.5993 (5)	0.2348 (4)	0.53138 (10)	0.0224 (6)	
H8A	0.6323	0.3286	0.5132	0.027*	
C9	0.5783 (5)	0.0934 (3)	0.50851 (10)	0.0213 (5)	
H9A	0.5488	0.0027	0.5284	0.026*	
C10	0.5971 (4)	0.0665 (3)	0.45512 (9)	0.0191 (5)	
C11	0.6283 (4)	−0.0843 (3)	0.43402 (10)	0.0199 (6)	
C12	0.6209 (4)	−0.1121 (3)	0.38335 (10)	0.0206 (6)	
H12A	0.6378	−0.2178	0.3708	0.025*	
C13	0.5890 (5)	0.0144 (3)	0.35161 (9)	0.0198 (5)	
C14	0.5639 (5)	0.1705 (3)	0.37128 (10)	0.0208 (6)	
C15	0.5659 (4)	0.1943 (3)	0.42198 (10)	0.0203 (6)	
H15A	0.5457	0.2995	0.4346	0.024*	
C16	0.5963 (5)	−0.1555 (4)	0.28128 (10)	0.0255 (6)	
H16A	0.5866	−0.1490	0.2453	0.038*	
H16B	0.4900	−0.2223	0.2941	0.038*	
H16C	0.7219	−0.2032	0.2904	0.038*	
C17	0.5316 (6)	0.4499 (4)	0.35512 (11)	0.0317 (7)	
H17A	0.5242	0.5236	0.3270	0.048*	
H17B	0.6509	0.4714	0.3739	0.048*	
H17C	0.4177	0.4657	0.3764	0.048*	

### Atomic displacement parameters (Å^2^)

**Table:**

	*U*^11^	*U*^22^	*U*^33^	*U*^12^	*U*^13^	*U*^23^
Br	0.04312 (19)	0.03241 (16)	0.01252 (15)	0.00224 (15)	−0.00131 (12)	−0.00345 (11)
O1	0.0530 (16)	0.0300 (11)	0.0157 (10)	−0.0028 (11)	0.0034 (10)	−0.0018 (9)
O2	0.0428 (14)	0.0333 (12)	0.0180 (11)	0.0038 (11)	−0.0085 (10)	0.0032 (9)
O3	0.0540 (16)	0.0227 (11)	0.0228 (11)	0.0026 (10)	0.0012 (10)	0.0005 (9)
O4	0.0377 (12)	0.0219 (9)	0.0123 (8)	−0.0006 (9)	−0.0012 (9)	−0.0026 (7)
O5	0.0456 (14)	0.0191 (10)	0.0125 (9)	−0.0002 (9)	−0.0034 (9)	0.0008 (8)
N1	0.0303 (13)	0.0223 (12)	0.0154 (11)	0.0032 (10)	0.0026 (10)	0.0010 (10)
C1	0.0200 (13)	0.0264 (13)	0.0135 (12)	0.0000 (12)	−0.0016 (12)	−0.0037 (10)
C2	0.0286 (15)	0.0251 (13)	0.0145 (13)	0.0015 (13)	0.0007 (13)	−0.0009 (10)
C3	0.0288 (14)	0.0309 (14)	0.0089 (11)	0.0029 (16)	−0.0014 (11)	−0.0035 (11)
C4	0.0282 (16)	0.0251 (13)	0.0207 (14)	0.0019 (13)	0.0019 (14)	−0.0027 (11)
C5	0.0298 (16)	0.0241 (14)	0.0200 (14)	0.0004 (13)	−0.0003 (13)	0.0022 (11)
C6	0.0255 (14)	0.0285 (14)	0.0150 (12)	0.0026 (15)	−0.0007 (11)	−0.0004 (11)
C7	0.0263 (15)	0.0280 (14)	0.0158 (13)	0.0005 (13)	−0.0018 (12)	−0.0008 (11)
C8	0.0258 (14)	0.0269 (13)	0.0146 (13)	−0.0019 (14)	0.0013 (12)	0.0002 (11)
C9	0.0252 (14)	0.0243 (13)	0.0144 (12)	0.0000 (12)	0.0004 (12)	0.0002 (10)
C10	0.0207 (12)	0.0234 (13)	0.0133 (12)	−0.0023 (13)	0.0001 (11)	−0.0006 (10)
C11	0.0227 (15)	0.0214 (13)	0.0155 (13)	−0.0002 (11)	0.0002 (11)	0.0025 (10)
C12	0.0267 (16)	0.0199 (12)	0.0150 (13)	−0.0012 (12)	0.0015 (12)	−0.0037 (10)
C13	0.0250 (14)	0.0229 (13)	0.0115 (11)	−0.0017 (12)	0.0004 (11)	−0.0024 (10)
C14	0.0253 (15)	0.0213 (13)	0.0159 (13)	−0.0019 (12)	0.0010 (11)	0.0023 (11)
C15	0.0232 (15)	0.0213 (13)	0.0163 (13)	−0.0007 (11)	−0.0001 (11)	−0.0022 (10)
C16	0.0371 (16)	0.0259 (14)	0.0135 (12)	0.0011 (14)	−0.0005 (14)	−0.0053 (10)
C17	0.056 (2)	0.0186 (14)	0.0210 (14)	−0.0006 (15)	0.0011 (13)	0.0006 (13)

### Geometric parameters (Å, °)

**Table:**

Br—C3	1.900 (3)	C7—C8	1.491 (4)
O1—C7	1.222 (4)	C8—C9	1.338 (4)
O2—N1	1.233 (4)	C8—H8A	0.9500
O3—N1	1.225 (4)	C9—C10	1.472 (4)
O4—C13	1.354 (3)	C9—H9A	0.9500
O4—C16	1.436 (3)	C10—C11	1.395 (4)
O5—C14	1.348 (4)	C10—C15	1.410 (4)
O5—C17	1.437 (4)	C11—C12	1.396 (4)
N1—C11	1.465 (4)	C12—C13	1.378 (4)
C1—C6	1.393 (4)	C12—H12A	0.9500
C1—C2	1.411 (4)	C13—C14	1.415 (4)
C1—C7	1.504 (4)	C14—C15	1.391 (4)
C2—C3	1.382 (4)	C15—H15A	0.9500
C2—H2A	0.9500	C16—H16A	0.9800
C3—C4	1.386 (4)	C16—H16B	0.9800
C4—C5	1.394 (4)	C16—H16C	0.9800
C4—H4A	0.9500	C17—H17A	0.9800
C5—C6	1.397 (4)	C17—H17B	0.9800
C5—H5A	0.9500	C17—H17C	0.9800
C6—H6A	0.9500		
			
C13—O4—C16	116.8 (2)	C10—C9—H9A	117.2
C14—O5—C17	117.1 (2)	C11—C10—C15	116.1 (2)
O3—N1—O2	123.1 (3)	C11—C10—C9	123.7 (3)
O3—N1—C11	118.9 (3)	C15—C10—C9	120.0 (2)
O2—N1—C11	118.0 (2)	C10—C11—C12	123.3 (3)
C6—C1—C2	119.5 (2)	C10—C11—N1	121.1 (2)
C6—C1—C7	123.8 (2)	C12—C11—N1	115.5 (2)
C2—C1—C7	116.6 (2)	C13—C12—C11	119.7 (3)
C3—C2—C1	118.9 (3)	C13—C12—H12A	120.2
C3—C2—H2A	120.6	C11—C12—H12A	120.2
C1—C2—H2A	120.6	O4—C13—C12	125.1 (2)
C2—C3—C4	122.2 (2)	O4—C13—C14	115.9 (2)
C2—C3—Br	118.8 (2)	C12—C13—C14	119.0 (2)
C4—C3—Br	119.1 (2)	O5—C14—C15	124.8 (3)
C3—C4—C5	118.9 (3)	O5—C14—C13	114.9 (2)
C3—C4—H4A	120.6	C15—C14—C13	120.2 (3)
C5—C4—H4A	120.6	C14—C15—C10	121.7 (3)
C4—C5—C6	120.2 (3)	C14—C15—H15A	119.1
C4—C5—H5A	119.9	C10—C15—H15A	119.1
C6—C5—H5A	119.9	O4—C16—H16A	109.5
C1—C6—C5	120.4 (2)	O4—C16—H16B	109.5
C1—C6—H6A	119.8	H16A—C16—H16B	109.5
C5—C6—H6A	119.8	O4—C16—H16C	109.5
O1—C7—C8	121.2 (3)	H16A—C16—H16C	109.5
O1—C7—C1	120.3 (3)	H16B—C16—H16C	109.5
C8—C7—C1	118.5 (3)	O5—C17—H17A	109.5
C9—C8—C7	119.1 (3)	O5—C17—H17B	109.5
C9—C8—H8A	120.5	H17A—C17—H17B	109.5
C7—C8—H8A	120.5	O5—C17—H17C	109.5
C8—C9—C10	125.5 (3)	H17A—C17—H17C	109.5
C8—C9—H9A	117.2	H17B—C17—H17C	109.5
			
C6—C1—C2—C3	0.4 (5)	C9—C10—C11—N1	−13.0 (5)
C7—C1—C2—C3	179.9 (3)	O3—N1—C11—C10	157.6 (3)
C1—C2—C3—C4	1.0 (5)	O2—N1—C11—C10	−25.0 (4)
C1—C2—C3—Br	−178.9 (3)	O3—N1—C11—C12	−26.6 (4)
C2—C3—C4—C5	−1.4 (5)	O2—N1—C11—C12	150.9 (3)
Br—C3—C4—C5	178.6 (3)	C10—C11—C12—C13	2.7 (5)
C3—C4—C5—C6	0.4 (5)	N1—C11—C12—C13	−173.1 (3)
C2—C1—C6—C5	−1.4 (5)	C16—O4—C13—C12	4.4 (5)
C7—C1—C6—C5	179.1 (3)	C16—O4—C13—C14	−176.6 (3)
C4—C5—C6—C1	1.0 (5)	C11—C12—C13—O4	178.8 (3)
C6—C1—C7—O1	−174.2 (3)	C11—C12—C13—C14	−0.2 (5)
C2—C1—C7—O1	6.3 (5)	C17—O5—C14—C15	8.8 (5)
C6—C1—C7—C8	7.0 (5)	C17—O5—C14—C13	−172.7 (3)
C2—C1—C7—C8	−172.5 (3)	O4—C13—C14—O5	0.6 (4)
O1—C7—C8—C9	3.7 (5)	C12—C13—C14—O5	179.7 (3)
C1—C7—C8—C9	−177.6 (3)	O4—C13—C14—C15	179.1 (3)
C7—C8—C9—C10	178.3 (3)	C12—C13—C14—C15	−1.8 (5)
C8—C9—C10—C11	161.5 (3)	O5—C14—C15—C10	179.9 (3)
C8—C9—C10—C15	−24.4 (5)	C13—C14—C15—C10	1.5 (5)
C15—C10—C11—C12	−2.9 (4)	C11—C10—C15—C14	0.8 (4)
C9—C10—C11—C12	171.4 (3)	C9—C10—C15—C14	−173.8 (3)
C15—C10—C11—N1	172.6 (3)		

### Hydrogen-bond geometry (Å, °)

**Table:**

*D*—H···*A*	*D*—H	H···*A*	*D*···*A*	*D*—H···*A*
C16—H16A···O5^i^	0.98	2.46	3.383 (3)	157
C17—H17B···O3^ii^	0.98	2.48	3.116 (4)	123

Symmetry codes: (i) −*x*+1, *y*−1/2, −*z*+1/2; (ii) *x*, *y*+1, *z*.

### Table 2 π-π hydrogen-bond geometry (Å)

Cg1 and Cg2 are the centroids of the C1–C6 and C10–C15 rings, respectively.

**Table:**

Cg···Cg	D···A
Cg1···Cg2^i^	3.7072 (18)
Cg1···Cg2^ii^	3.6326 (18)

Symmetry codes: (i) -1/2+x, 1/2-y, 1-z; (ii) 1/2+x, 1/2-y, 1-z.
